# CCR5 limits cortical viral loads during West Nile virus infection of the central nervous system

**DOI:** 10.1186/s12974-015-0447-9

**Published:** 2015-12-15

**Authors:** Douglas M. Durrant, Brian P. Daniels, TracyJo Pasieka, Denise Dorsey, Robyn S. Klein

**Affiliations:** Department of Medicine, Washington University School of Medicine, St Louis, MO 63110 USA; Department of Pathology & Immunology, Washington University School of Medicine, St Louis, MO 63110 USA; Department of Anatomy & Neurobiology, Washington University School of Medicine, St Louis, MO 63110 USA

**Keywords:** CCR5, Viral encephalitis, Blood-brain barrier, Cerebral cortex, T cell, Macrophage

## Abstract

**Background:**

Cell-mediated immunity is critical for clearance of central nervous system (CNS) infection with the encephalitic flavivirus, West Nile virus (WNV). Prior studies from our laboratory have shown that WNV-infected neurons express chemoattractants that mediate recruitment of antiviral leukocytes into the CNS. Although the chemokine receptor, CCR5, has been shown to play an important role in CNS host defense during WNV infection, regional effects of its activity within the infected brain have not been defined.

**Methods:**

We used CCR5-deficient mice and an established murine model of WNV encephalitis to determine whether CCR5 activity impacts on WNV levels within the CNS in a region-specific fashion. Statistical comparisons between groups were made with one- or two-way analysis of variance; Bonferroni’s post hoc test was subsequently used to compare individual means. Survival was analyzed by the log-rank test. Analyses were conducted using Prism software (GraphPad Prism). All data were expressed as means ± SEM. Differences were considered significant if *P* ≤ 0.05.

**Results:**

As previously shown, lack of CCR5 activity led to increased symptomatic disease and mortality in mice after subcutaneous infection with WNV. Evaluation of viral burden in the footpad, draining lymph nodes, spleen, olfactory bulb, and cerebellum derived from WNV-infected wild-type, and CCR5^−/−^ mice showed no differences between the genotypes. In contrast, WNV-infected, CCR5^−/−^ mice exhibited significantly increased viral burden in cortical tissues, including the hippocampus, at day 8 post-infection. CNS regional studies of chemokine expression via luminex analysis revealed significantly increased expression of CCR5 ligands, CCL4 and CCL5, within the cortices of WNV-infected, CCR5^−/−^ mice compared with those of similarly infected WT animals. Cortical elevations in viral loads and CCR5 ligands in WNV-infected, CCR5^−/−^ mice, however, were associated with decreased numbers of infiltrating mononuclear cells and increased permeability of the blood-brain barrier.

**Conclusions:**

These data indicate that regional differences in chemokine expression occur in response to WNV infection of the CNS, and that cortical neurons require CCR5 activity to limit viral burden in this brain region.

## Background

Infection with the encephalitic flavivirus, West Nile virus (WNV), is the leading cause of domestically acquired arboviral disease in the USA [1]. Acute infectious syndromes after infection with WNV include a self-limited febrile illness, West Nile fever (WNF), or more severe neuroinvasive diseases (WNND), including meningitis, encephalitis, or flaccid paralysis. The entry of virus-specific T cells into the CNS parenchyma is essential for viral clearance and survival in both human and murine subjects with WNV encephalitis [2–6]. Indeed, the increased incidence of WNV neuroinvasive disease in patients on anti-T cell therapies [5, 7] and in mice with T cell deficiencies [4, 8–10] indicates that the clearance of WNV within the CNS relies heavily on cell-mediated immune responses that promote the CNS entry and effector functions of CD8^+^ T cells [11, 12]. While studies indicate that CNS regions differ in the extent of inflammatory infiltrates during viral encephalitis [13–16], regional differences in expression of guidance cues that promote T cell entry have not been established. These guidance cues, combined with the tightly controlled egress of leukocytes from the perivascular sites, coordinate the migration of leukocyte subsets for protective and pathologic purposes.

In most tissues, leukocyte recruitment is orchestrated by a series of coordinated leukocyte-endothelial interactions involving several families of molecular regulators including selectins, integrins, and chemokines [17, 18]. Chemokines are a superfamily of over 50 structurally homologous chemotactic, heparin binding, secreted proteins with their target cell specificity conferred by pertussis toxin (PTX) sensitive, G_αi_-coupled seven transmembrane glycoprotein chemokine receptors. Of interest, CXCL12 and its receptors are believed to most resemble the ancestral chemokine-receptor pair [19] suggesting that CXC chemokines are evolutionarily older than CC chemokines. In published studies, we have determined that upregulation of proinflammatory chemokines during WNV encephalitis may occur in a region-specific fashion [2, 20]. For example, cerebellar expression of CXCL10 is required for viral clearance of this brain region by CXCR3-expressing, virus-specific CD8^+^ T cells [20]. Differences in regional chemokine expression may thus determine the spatial patterns of leukocyte trafficking, leading to variability in viral clearance and immunopathology between CNS regions.

CCL3–5, chemokines that all bind the chemokine receptor CCR5, are strongly induced in the CNS after WNV infection [2, 21–23]. Monocytes, NK and T cells express CCR5 and targeted deletion of CCR5 in B6129PF2 mice is associated with depressed leukocyte trafficking, increased viral burden and enhanced mortality [21]. Similarly, homozygosity for CCR5Δ32, a nonfunctional variant of chemokine receptor CCR5, is markedly increased among symptomatic WNV-seropositive patients [22, 24]. In the current study, we examined the role of CCR5 in C57BL/6 mice during WNV infection, focusing on CNS region-specific effects. We found that CCR5 is required for virologic control specifically within the CNS cortex. This finding was associated with a significant decrease in immune cell infiltrates, increased blood-brain barrier (BBB) permeability, and elevated levels of CCR5 ligands in WNV-infected CCR5^−/−^ compared with WT mice. These data suggest that increased viral replication within the CNS modulates BBB function and support the notion that nonredundancy in chemokine-mediated inflammation among CNS regions may be due to evolutionary mechanisms.

## Methods

### Murine model of WNV encephalitis

Eight-week-old C57BL/6 wild-type mice were obtained commercially (Jackson Laboratories). Congenic *ccr5*^−/−^ mice were also commercially obtained (Jackson Laboratories, stock number 005427) and bred in the animal facilities at the Washington University School of Medicine. All animals were housed under pathogen-free conditions in the animal facilities of the Washington University School of Medicine. All experiments were performed in compliance with Washington University animal studies guidelines and comply with “Animal Research: Reporting In Vivo Experiments (ARRIVE) guidelines.” Mice were inoculated subcutaneously via footpad injection (50 μl) with 100 PFU of WNV as previously described [25]. WNV strain 3000.0259 was isolated in New York in 2000 [26] and passaged once in C6/36 cells. Viral titers were measured by plaque assay on BHK21-15 cells as previously described [27]. Cortical, cerebellar, and brainstem regions of the CNS were dissected based on visual information, such as differences in color of adjacent tissues, and on the natural anatomical boundaries of certain regions present in the brain. Clinical disease was monitored and scored as previously described [28]. The designation for the clinical scores is as follows: 1 ruffled fur/hunched, 2 paresis/difficulty walking, 3 paralysis, 4 moribund, and 5 dead.

### BBB permeability assay

At day 5 and 8 after WNV infection, mice were injected intraperitoneally (IP) with sodium fluorescein dye (100 mg/ml) as previously described [29]. After 45 min, mice were perfused, serum was collected, and CNS tissues were harvested. Both the serum and tissue homogenates were incubated overnight at 4 °C in 2 % trichloroacetic acid at 1:1 dilution to precipitate protein and then the supernatants were neutralized in equal volumes of borate buffer. Fluorescence emission at 538 nm was determined using a microplate reader Synergy™ H1 and Gen5™ software (BioTek Instruments, Inc.). Fluorescence concentration was calculated from a standard curve, and tissue fluorescence values were normalized to serum fluorescence values from identical mice.

### Chemokine analysis

The chemokine Bio-Plex assay was performed on tissue samples from mice at day 8 after infection from WNV-infected mice. Tissue was homogenized following extensive cardiac perfusion with PBS and was analyzed using a 6-plex Luminex assay (Millipore) followed by analysis on a Bio-Plex 200 (Bio-Rad). Concentrations of chemokines were normalized to total protein levels.

### Leukocyte isolation

Cells were isolated from the CNS of WT and CCR5^−/−^ mice at day 6 and 8 after WNV infection. Following cell count and viability analysis, cells were stained with fluorescently conjugated antibodies to CD4, CD8β, CD11b, and CD45 as previously described [30]. Samples were analyzed following staining using a LSRII flow cytometer (Beckton Dickinson) to collect up to 30,000 events in a broad gate defined by forward- and side-scatter attributes. The absolute count of respective leukocyte subsets was calculated based on the percent positive cells from data analysis, which was performed using FlowJo software (Tree Star).

### Immunohistochemistry

Brain tissues were perfused with 4 % paraformaldehyde and isolated for frozen sections. Tissue sections were permeabilized and blocked in 0.1 % Triton X-100 and 10 % goat serum, followed by incubation with primary antibodies CD-3 1:200 (Dako) and WNV antigen 1:100 (Diamond lab) or CD-3 1:200 (Dako), CD31 1:20 (BD) and endomucin 1:200 (eBioscience/Affymetrix) overnight at 4 °C. Primary antibodies were detected with goat anti-rabbit Alexa Fluor 488 (CD3), goat anti-rat Alexa Fluor 555 (WNV antigen) and goat anti-rat Alexa Fluor 555 (CD31, endomucin) followed by nuclear DAPI counterstaining. Tissues were then washed and examined by confocal microscopy.

### Statistical analysis

Statistical comparisons between groups were made with one- or two-way analysis of variance; Bonferroni’s post hoc test was subsequently used to compare individual means. Survival was analyzed by the log-rank test. Analyses were conducted using Prism software (GraphPad Prism). All data were expressed as means ± SEM. Differences were considered significant if *P* ≤ 0.05.

## Results

### CCR5-deficient mice exhibit higher cortical viral loads with increased BBB permeability

**Fig. 1:**
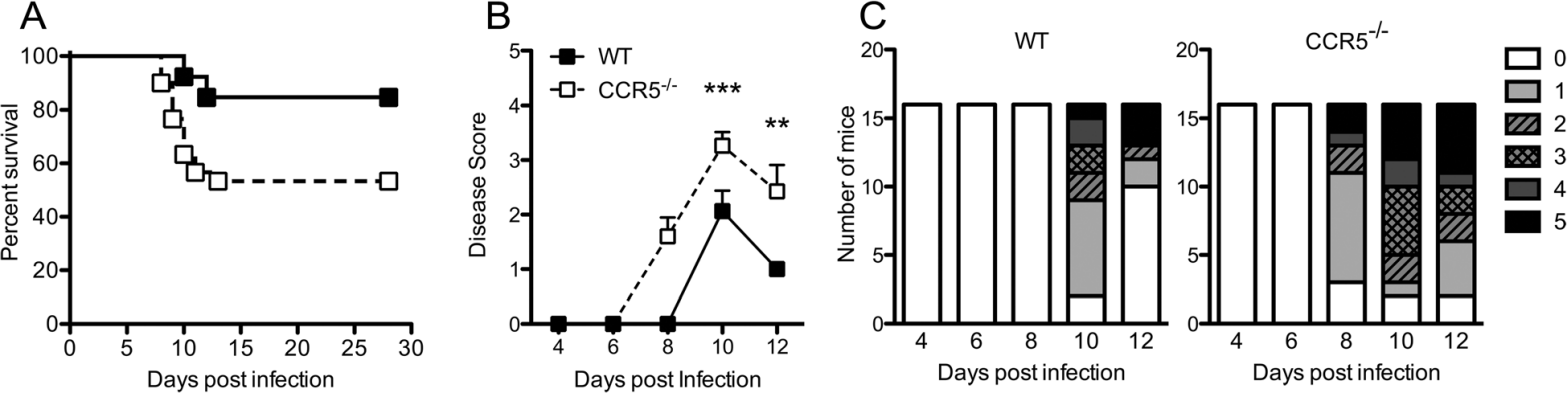
CCR5 is necessary to reduce WNV-associated disease and mortality. WT and CCR5^−/−^ mice were infected with 10^2^ PFU of WNV via the subcutaneous route. **a** Survival was monitored for 28 days after infection. *n* = 30 mice, *P = 0.0503 (log-rank test). **b** Clinical scores were monitored daily for 12 days after infection. The designation for the clinical scores is as follows: 1 ruffled fur/hunched; 2 altered gait, slow movement; 3 not moving but responsive; 4 moribund; and 5 death. **c** Individual clinical scores are shown at each time point for WT (left) and CCR5^−^/^−^ (right) mice. *Asterisks* indicate values that are statistically significant (**P < 0.005, ***P < 0.001)

**Fig. 2:**
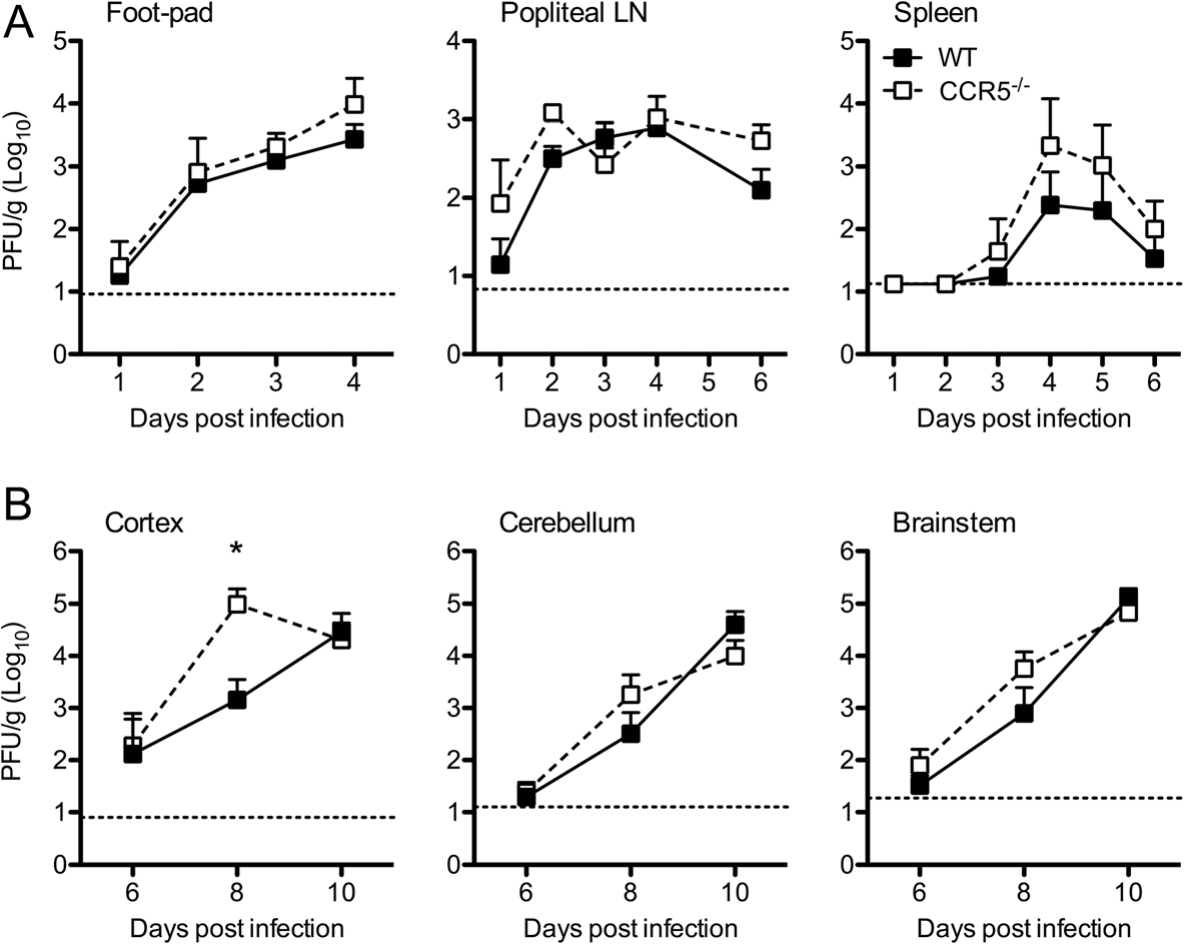
CCR5 restricts WNV replication in cortical tissues of the CNS. WT and CCR5^−/−^ mice were infected via footpad with 10^2^ PFU of WNV. **a** Viral burden within the footpad, draining lymph node (popliteal LN), and spleen of WT and CCR5^−/−^ mice were measured at indicated days post-infection. **b** WNV levels within the cortex, cerebellum, and brainstem were measured at days 6, 8, and 10 post-infection. Tissue homogenates were analyzed for viral burden by infectious plaque assay. Data are shown as the mean PFU per gram of tissue from 6 to 10 mice per time point. *Dotted lines* denote the limit of detection of the respective assays. *Asterisks* indicate values that are statistically significant (*P < 0.05)

In prior studies, mice with targeted deletion of CCR5 (B6129PF2 background) exhibited 100 % lethality after subcutaneous inoculation with WNV-NY (strain NY9935262; 10^4^ ffu), which was attributed to the decreased CNS entry of all leukocyte subpopulations including macrophages, T and NK cells [21]. In this study, mortality due to WNV infection was not significantly enhanced by increases in WNV inoculum beyond 10^2^ focus-forming units. To determine whether CCR5-deficient animals (C57BL/6 background) exhibited similar survival effects, 8-week-old, C57BL/6 wild-type (WT), and CCR5-deficient mice were infected subcutaneously via footpad inoculation with 10^2^ plaque-forming units (PFU) of strain WNV-NY99 and followed for severity of disease and survival. WNV-infected CCR5^−/−^ mice exhibited enhanced mortality (Fig. 1a, WT 20 %; CCR5^−/−^ 50 %) and significantly higher clinical disease severity scores (Fig. 1b, c), compared with similarly infected WT animals. Analysis of viral loads in the footpad and peripheral organs did not reveal differences between the two genotypes at any time points after WNV infection (Fig. 2a). In contrast, analyses of CNS tissues detected significantly higher viral loads in the cortices, but not within the brainstem or cerebella, of WNV-infected CCR5^−/−^ compared with WT mice (Fig. 2b). Of note, because of the difference in survival observed between genotypes, the CCR5-deficient mice assessed at day 10 do not include those that have succumbed to viral encephalitis and will naturally include those with lower viral titers that do survive. WT animals exhibit more survival and generally exhibit peak viral loads at days 8–10, which undergo clearance thereafter [3, 12], leading to similar viral loads at this later time point. These data suggest that CCR5 is critical for virologic control specifically within the cortex during WNV encephalitis.

**Fig. 3:**
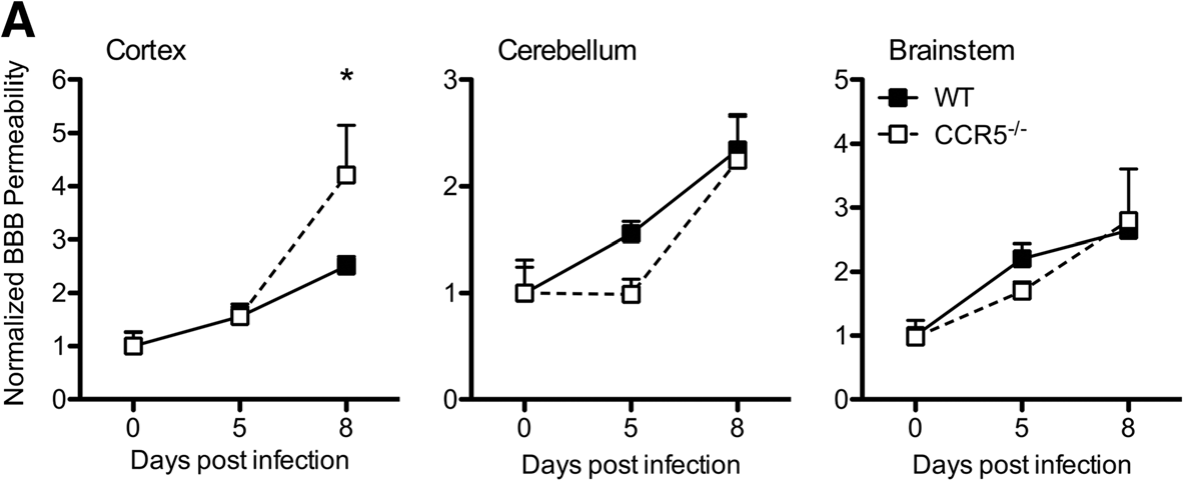
BBB permeability increases within the cortices of the WNV-infected CCR5^−/−^ CNS. **a** Following inoculation with 10^2^ PFU of WNV in the footpad, WT and CCR5^−/−^ mice were assessed for BBB sodium fluorescein permeability in different CNS regions on the indicated days post-infection. BBB permeability was determined by the accumulation of sodium fluorescent dye in CNS tissues after intraperitoneal administration. Data are shown as mean fluorescence normalized to the mean value for uninfected animals ± SEM for four mice per time point and are representative of two independent experiments. *Asterisks* indicate values that are statistically significant (*P < 0.05)

In recently published studies, we demonstrated that detection of WNV by host tissues induces local innate immune cytokine expression by CNS endothelium, regulating BBB structure and function [29]. Given the increased viral load in the cortices of CCR5^−/−^ mice compared with similarly infected WT, we performed fluorometric assessment of BBB permeability following the intraperitoneal (IP) administration of sodium fluorescein. Cerebellar and brainstem tissues derived from WNV-infected WT and CCR5^−/−^ mice exhibited similar increases in extravasation of sodium fluorescein at 5 and 8 days post-infection (Fig. 3). However, similar analyses of cortical tissues revealed a significant increase in BBB permeability at 8 days post-infection in CCR5^−/−^ mice compared with WT controls (Fig. 3). These findings are consistent with previous data linking higher viral loads with worsened BBB stability [31, 32].

### CCR5-deficient mice exhibit increased chemokines within the cortex with less inflammation

**Fig. 4:**
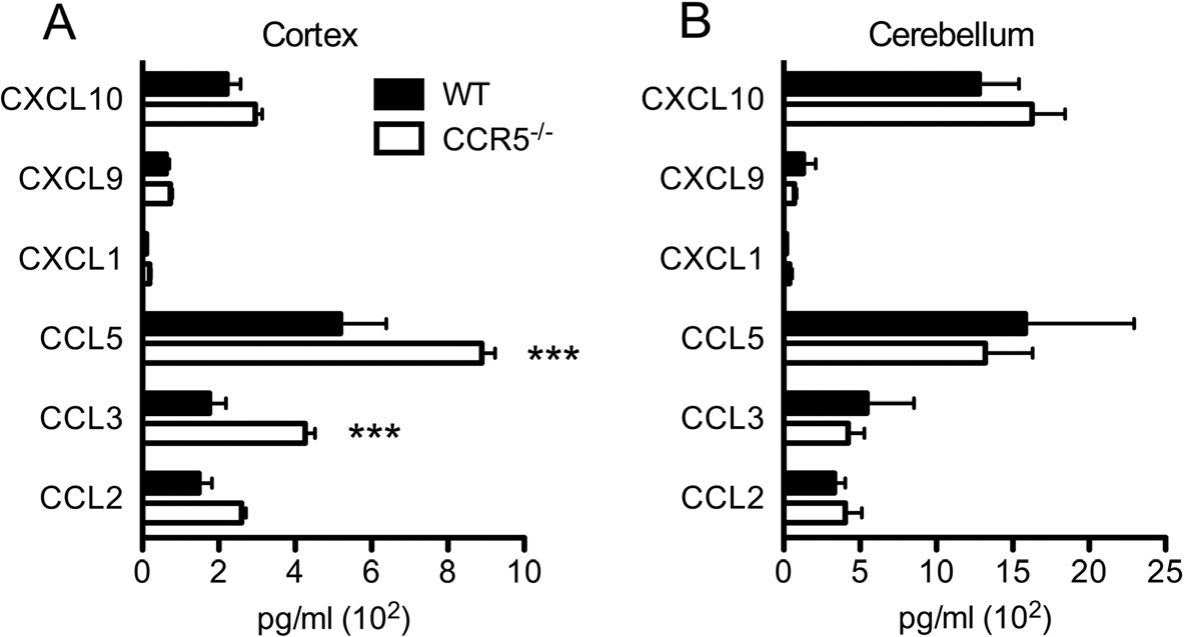
CCR5 ligands are increased within the cortical region of the CCR5^−/−^ CNS during WNV infection. Expression of chemotactic cytokines was measured in the (**a**) cortical and (**b**) cerebellar regions of the CNS harvested from WNV-infected WT and CCR5^−/−^ mice at day 8 post-infection by Bio-Plex assay. Results represent means ± SEM of five mice per group. *Asterisks* indicate values that are statistically significant (***P < 0.001)

**Fig. 5:**
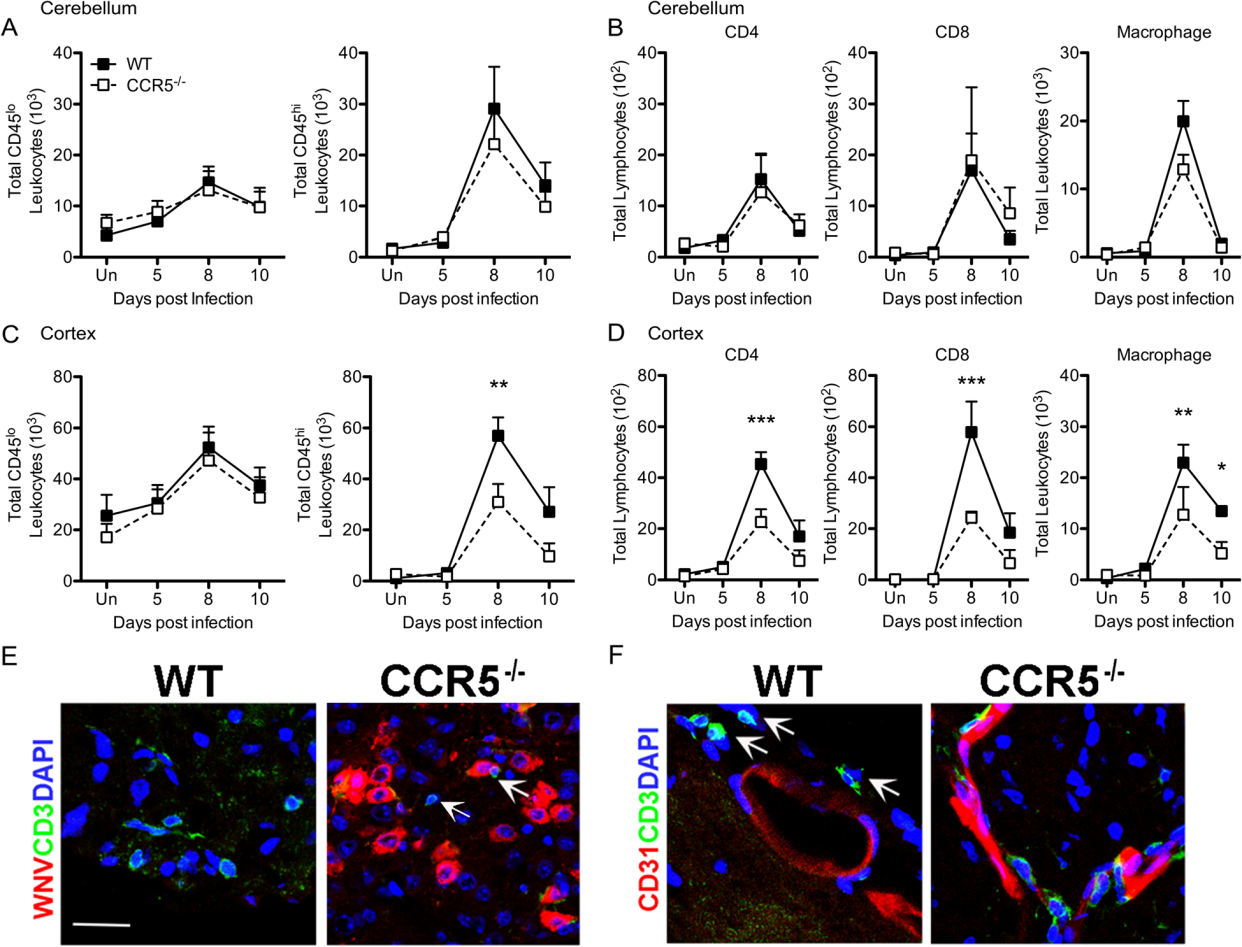
CCR5 is necessary for recruitment of mononuclear cells to the cortical region of the WNV-infected CNS. The immunophenotype of brain leukocytes and their locations within the cortex were determined in WNV-infected WT and CCR5^−/−^ mice. Mice were infected with 10^2^ PFU of WNV in the footpad. The cerebellum (**a**, **b**) and cortex (**c**, **d**) were harvested and analyzed by flow cytometry at the indicated days after infection. In separate infections, cortical tissues from WNV-infected WT and CCR5^−/−^ mice were examined for locations of CD3+ cells with respect to WNV antigen or the vasculature at day 8 post infection. **a**, **c** The numbers of CD45^lo^ and CD45^hi^ leukocyte populations were determined at the indicated time points after infection. **b**, **d** The numbers of CD4^+^, CD8^+^, and CD11b^+^ (macrophage) populations from the CD45^hi^ leukocyte population were determined at the indicated time points after infection. Results represent the means ± SEM of at least four mice per group. *Asterisks* indicate values that are statistically significant (*P < 0.05, **P < 0.01). **e** Detection of WNV antigen (red) and CD3+ T cells (green). Nuclei were counterstained (blue). **f** Detection of CD31+ endothelial cells (red) and CD3+ T cells (green). Images are representative of 10 images per mouse for 3 mice/genotype. In all images, *arrows* indicate CD3+ cells. Scale bar = 25 μm

In prior studies, elevated viral loads within the CNS of WNV-infected mice were also linked to increased expression of chemoattractant cytokines [2, 33]. Consistent with this, at time points when viral loads in the cortices of WNV-infected CCR5^−/−^ mice are highest, levels of CCR5 ligands, CCL3 and CCL5, were significantly elevated compared with cortical tissues of similarly infected WT mice (Fig. 4a). Moreover, there were no differences between the genotypes in chemokine expression levels in the cerebella of WNV-infected mice (Fig. 4b). Given the increase in CCR5 chemoattractants and increased BBB permeability, we wondered whether WNV-infected CCR5 mice would exhibit increased parenchymal entry of mononuclear cells. To address this, we performed flow cytometric analyses of infiltrating mononuclear cells within the cortices and cerebella of WNV-infected WT versus CCR5^−/−^ mice on days 5, 8, and 10 post-infection. Inflammatory infiltrates, CD45^hi^ leukocytes, within the cerebella of WNV-infected WT and CCR5-deficient mice peaked at day 8 post-infection and exhibited similar total numbers of CD45^lo^ and CD45^hi^ mononuclear cells (Fig. 5a). In contrast, total numbers of infiltrating CD45^hi^ leukocytes were significantly higher in cortical tissues of WNV-infected WT mice, compared with their CCR5^−/−^ counterparts, on day 8 post-infection (Fig. 5c). Although similar numbers of CD45^hi^ infiltrating leukocytes, comprised of CD4^+^ and CD8^+^ (lymphocytes) and CD11b^+^ (macrophages) were present between WT and CCR5^−/−^ mice in the cerebellar tissues on day 8 following WNV infection (Fig. 5b), the total number of CD45^hi^ CD4^+^ and CD8^+^ lymphocytes was significantly decreased in the cortices of CCR5-deficient, WNV-infected mice on day 8 post-infection (Fig. 5d). CD45^hi^ CD11b^+^ leukocytes (macrophages) were decreased in both genotypes on 10 post-infection (Fig. 5d). Immunohistochemical analyses of T cell infiltration within the cortices of WNV-infected, WT and CCR5^−/−^ mice at day 8 post-infection revealed T cell infiltration with little WNV antigen in WT hosts. In contrast, low numbers of parenchymal T cells were detected within CCR5-deficient hosts, despite increased detection of WNV antigen compared with WT animals (Fig. 5e). Moreover, vessel-associated T cells were increased in CCR5^−/−^ mice, while parenchymal T cells predominated in WT animals (5F). Taken altogether, these data indicate that CCR5 is not required for antiviral, cell-mediated immunity in the periphery. In the CNS, however, CCR5 is required for the entry of leukocytes specifically into cortex.

## Discussion

While CCR5 has been shown to be critical for survival of WNV-infected mice, region-specific roles for this receptor within the CNS has not been previously described. Here, we demonstrate that targeted deletion of CCR5 leads to loss of virologic control specifically within cortical tissues of the CNS, which also exhibit enhanced BBB permeability and elevated levels of CCR5 ligands, CCL3 and CCL5. Despite these latter findings, WNV-infected CCR5^−/−^ mice showed decreased infiltration of all mononuclear cells into cortical regions compared with similarly infected WT animals. These data suggest region-specific roles for CCR5 ligands within the virally infected CNS.

Although mortality of WNV-infected, CCR5^−/−^ mice were only moderately increased, symptomatic disease was significantly enhanced. This is reminiscent of findings in patients with the Δ32CCR5 mutation, which exhibit increase in symptomatic diseases after WNV infection, but no increase in mortality [22, 34]. In previous studies using CCR5^−/−^ mice on a B6129PF2 background, infection with WNV-NY99 led to uniform mortality. The severe phenotype observed in CCR5-deficient mice may be due to strain related variations in inflammatory responses between C57BL/6 versus mixed background (B6129PF2) strains [35].

Increased symptoms of encephalitis in CCR5-deficient mice in our study were associated with increased cortical viral loads. This region-specific effect is consistent with the other reports of regional differences in expression of inflammatory molecules during viral infections of the CNS [33, 36]. The lack of effect of CCR5-deficiency on viral loads in the periphery or in other CNS regions suggests that CCR5^+^ antiviral lymphocytes are specifically required for virologic control within the cortex. Of note, adoptive transfer of CCR5-sufficient cells into WNV-infected, CCR5-deficient mice were previously shown to control viral replication and improve survival [21], which may have been due to improved virologic control in the forebrain. This is similar to the findings regarding the role of CXCR3, which is dispensable for the control of viral infection in the periphery and in most CNS compartments but required for CD8 T cell-mediated antiviral responses specifically within the cerebellum. That evolutionary older CXC chemokines would be important for virologic control in the hindbrain while CC chemokines, which evolved with adaptive immunity, would play a bigger antiviral role in the forebrain is quite accordant [19, 37, 38]. Recent studies also indicate that activated microglia in the setting of WNV encephalitis express proinflammatory cytokines and chemokines [39]. Further studies evaluating regional differences in chemoattractant responses of various neural cell types during viral infections should yield important insights regarding how viruses differentially impact CNS antiviral responses.

We previously showed that clearance of virus is associated with prompt resolution of encephalitis, with decreased numbers of infiltrating T cells [40]. Consistent with this, WT mice exhibited increased parenchymal T cells and low levels of WNV antigen at day 8 post-infection followed by decreased numbers of CNS-infiltrated leukocytes at day 10 post-infection. CNS infiltrating T cells and macrophages express cytokines, such as interferon (IFN)-*γ*, tumor necrosis factor (TNF)-α, and interleukin (IL)-1β, may induce disruption of interendothelial cell tight junctions (TJs) via activation of the GTPase RhoA [11, 29]. The resulting increase in BBB permeability is associated with the migration of mononuclear cells into the CNS parenchyma, with enhanced viral clearance [40]. In our study, despite the increase in BBB permeability observed in cortical tissues of WNV-infected, CCR5^−/−^ mice, significantly fewer leukocytes trafficked into this CNS region. The increase in BBB permeability in the setting of high MOIs of WNV is likely due to effects of inflammasome activation, which disrupts tight junction integrity via effects of IL-1β [29]. In addition, high MOIs of WNV which induce necrotic cell death and cause cells to release immunogenic factors as they die including inflammatory cytokines like HMGB1, while low MOIs induce apoptosis, which is typically non-immunogenic [41]. This is consistent with prior studies showing that pattern recognition receptor activation in the presence of WNV in the BBB endothelium results in cytokine-dependent modulation of Rho GTPase signaling, exerting regulatory control over BBB permeability and TJ integrity [29]. Thus, it is possible that the high viral loads within the cortices of WNV-infected, CCR5^−/−^ mice induce viral sensing mechanisms, such as activation of Toll-like receptor 3 [29, 42], that promote loss of BBB integrity.

## Conclusions

In conclusion, our data identify CCR5 as critically important for cell-mediated immunity during cortical infections with neurotropic virus. Loss of CCR5 results in decreased ability to recruit antiviral mononuclear cells specifically into WNV-infected, cortical tissues, which are essential for virologic control. The data also raise new questions regarding the effects of high CNS viral loads on BBB permeability and the differential expression of chemokines within brain regions during viral infections of the CNS.
